# iPSC-derived NK cells expressing high-affinity IgG Fc receptor fusion CD64/16A to mediate flexible, multi-tumor antigen targeting for lymphoma

**DOI:** 10.3389/fimmu.2024.1407567

**Published:** 2024-07-19

**Authors:** Kate J. Dixon, Kristin M. Snyder, Melissa Khaw, Robert Hullsiek, Zachary B. Davis, Anders W. Matson, Soheila Shirinbak, Bryan Hancock, Ryan Bjordahl, Martin Hosking, Jeffrey S. Miller, Bahram Valamehr, Jianming Wu, Bruce Walcheck

**Affiliations:** ^1^ Department of Veterinary and Biomedical Sciences, University of Minnesota, St. Paul, MN, United States; ^2^ Division of Hematology, Oncology, and Transplantation, Department of Medicine, University of Minnesota, Minneapolis, MN, United States; ^3^ Fate Therapeutics, San Diego, CA, United States; ^4^ Center for Immunology, University of Minnesota, Minneapolis, MN, United States; ^5^ Masonic Cancer Center, University of Minnesota, Minneapolis, MN, United States; ^6^ Stem Cell Institute, University of Minnesota, Minneapolis, MN, United States

**Keywords:** natural killer (NK) cell, antibody, ADCC - antibody-dependent cellular cytotoxicity, immunotherapy, cancer

## Abstract

**Introduction:**

NK cells can mediate tumor cell killing by natural cytotoxicity and by antibody-dependent cell-mediated cytotoxicity (ADCC), an anti-tumor mechanism mediated through the IgG Fc receptor CD16A (FcγRIIIA). CD16A polymorphisms conferring increased affinity for IgG positively correlate with clinical outcomes during monoclonal antibody therapy for lymphoma, linking increased binding affinity with increased therapeutic potential via ADCC. We have previously reported on the FcγR fusion CD64/16A consisting of the extracellular region of CD64 (FcγRI), a high-affinity Fc receptor normally expressed by myeloid cells, and the transmembrane/cytoplasmic regions of CD16A, to create a highly potent and novel activating fusion receptor. Here, we evaluate the therapeutic potential of engineered induced pluripotent stem cell (iPSC)-derived NK (iNK) cells expressing CD64/16A as an “off-the-shelf”, antibody-armed cellular therapy product with multi-antigen targeting potential.

**Methods:**

iNK cells were generated from iPSCs engineered to express CD64/16A and an interleukin (IL)-15/IL-15Rα fusion (IL-15RF) protein for cytokine independence. iNK cells and peripheral blood NK cells were expanded using irradiated K562-mbIL21–41BBL feeder cells to examine in *in vitro* and *in vivo* assays using the Raji lymphoma cell line. ADCC was evaluated in real-time by IncuCyte assays and using a xenograft mouse model with high circulating levels of human IgG.

**Results:**

Our data show that CD64/16A expressing iNK cells can mediate potent anti-tumor activity against human B cell lymphoma. In particular, (i) under suboptimal conditions, including low antibody concentrations and low effector-to-target ratios, iNK-CD64/16A cells mediate ADCC, (ii) iNK-CD64/16A cells can be pre-loaded with tumor-targeting antibodies (arming) to elicit ADCC, (iii) armed iNK-CD64/16A cells can be repurposed with additional antibodies to target new tumor antigens, and (iv) cryopreserved, armed iNK-CD64/16A are capable of sustained ADCC in a tumor xenograft model under saturating levels of human IgG.

**Discussion:**

iNK-CD64/16A cells allow for a flexible use of antibodies (antibody arming and antibody targeting), and an “off-the-shelf” platform for multi-antigen recognition to overcome limitations of adoptive cell therapies expressing fixed antigen receptors leading to cancer relapse due to antigen escape variants.

## Graphical Abstract

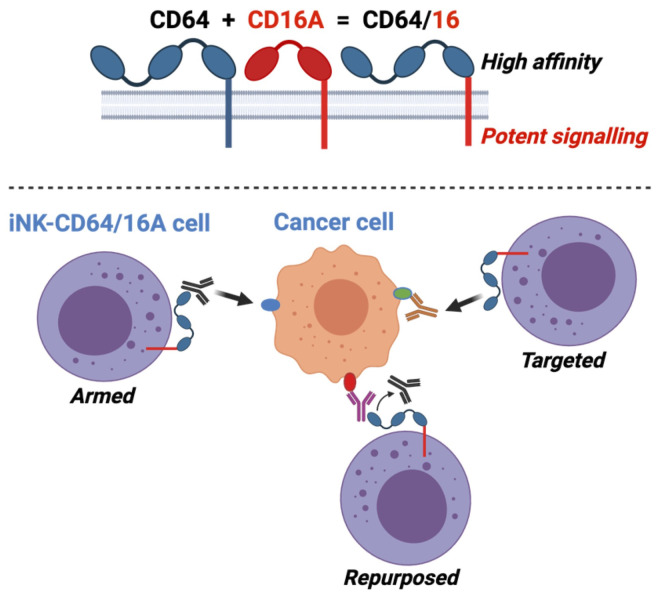

## Introduction

Current FDA-approved chimeric antigen receptor (CAR) T cell-based therapies have shown promise in treating several hematological malignancies (1, 2). However, hurdles with this autologous adoptive cell therapy (ACT) include long manufacturing processes coupled with the high cost of CAR engineering patient cells on an individual basis (3, 4). Many cancer patients also suffer from lymphopenia due to chemotherapy-based treatments, thus problems collecting adequate numbers of functional T cells to engineer may limit therapeutic efficacy (5, 6). Of particular importance is that current FDA-approved CAR-based therapies target only one tumor antigen. Increasing evidence indicates that this drives antigen loss and relapse by antigen-negative tumor cells (2). CAR-T cell clinical trials for B-cell acute lymphoblastic leukemia (B-ALL) show a range of CD19-negative relapse rates of 10–25%, with pediatric trials having a higher antigen-negative relapse rate (5, 7–11). A study of pediatric B-ALL found that 18 months after anti-CD19 CAR T therapy, the relapse-free survival was over 65%; however, of the patients who relapsed, 74% were CD19-negative (12). Antigen escape was also identified as the mechanism of relapse in 20–28% of CAR T cell-treated B cell lymphoma patients (13–15), with one study concluding that the path forward in CAR T cell therapy needed to include multiantigen targeting (15). This emphasizes that while CAR T therapy has improved cancer immunotherapy, addressing antigen escape is a critical next step.

Given the challenges with T cell ACT, other cell types are being investigated for cancer immunotherapies. Natural killer (NK) cells, cytotoxic lymphocytes that are part of the innate immune system, express an array of nonclonotypic inhibitory and activation receptors for the recognition of ligands and antigens on tumor cells (16). Upon their activation, NK cells release cytolytic factors as well as proinflammatory cytokines that modulate innate and adaptive immune cells (17). Given these anti-tumor effector functions, NK cell therapies are being actively investigated in clinical trials, with a particular focus on allogeneic ACT. A key advantage of NK cells is that they can be directed to diverse tumor antigens using therapeutic mAbs to mediate antibody-dependent cell-mediated cytotoxicity (ADCC) (18).

An ever-increasing arsenal of FDA-approved antibodies for cancer immunotherapy provides NK cells with multi-specificity in tumor antigen targeting to overcome antigen escape. Hence, optimizing ADCC is a critical focus in improving NK cell-based therapies. ADCC by NK cells is exclusively mediated by CD16A (FcγRIIIA) (18, 19). Leveraging the endogenously expressed CD16A receptor, however, has several limitations that reduce ADCC potency (20). CD16A is dynamically regulated at the cell membrane upon NK cell activation by a disintegrin and metalloproteinase 17 (ADAM17) (21–23). This membrane-associated protease rapidly cleaves CD16A, decreasing its surface density during ADCC, resulting in reduced CD16A avidity as a negative feedback process (20). CD16A binding avidity regulates cell-cell contacts and forming an immunological synapse (24). Moreover, CD16A downregulation occurs in the tumor microenvironment where NK cells are often found to be CD16A low or negative (22, 25–27). Although normally expressed as a low affinity IgG Fc receptor, allelic variations of CD16A have been identified that enhance IgG binding affinity and correspond with higher response rates in patients treated with therapeutic mAbs (28, 29). This evidence thus supports that increasing CD16A affinity and avidity to therapeutic mAbs would improve the ADCC potency of NK cell-based ACT.

Our goal is to develop an “off-the-shelf” NK cell therapy with enhanced ADCC potency to target multiple tumor antigens expressed by hematologic malignancies to reduce antigen negative relapse. We have previously described an FcγR fusion consisting of the extracellular portion of CD64 (FcγRI) combined with the transmembrane and intracellular regions of CD16A, referred to as CD64/16A (30, 31). Due to its high-affinity state, NK cells expressing CD64/16A can be armed with therapeutic mAbs to significantly enhance ADCC (30, 32). In addition to higher binding affinity, CD64 is not an ADAM17 substrate and CD64/16A is not cleaved from the cell surface upon cell activation (30).

Here, we demonstrate that iPSC-derived NK (iNK) cells expressing CD64/16A when in the presence of, or armed with, therapeutic mAbs mediate potent ADCC against B cell lymphoma cells. Antibody-armed iNK-CD64/16A cells maintain antibody targeting over several rounds of killing, demonstrating durability of antibody-directed killing. Further, by switching therapeutic antibodies, iNK-CD64/16A can be armed to target multiple tumor antigens to address antigen escape. In *in vitro* studies and using a modified tumor xenograft model, cryopreserved rituximab-armed iNK-CD64/16A cells exhibit potent ADCC in the presence of saturating human IgG. These findings demonstrate that iNK-CD64/16A cells are a potential “off-the-shelf” therapy to treat B cell lymphoma wherein overcoming antigen escape is critical.

## Methods

### Cell culture

iPSCs were generated by a transgene-free approach in feeder-free culture conditions (33). They were engineered by sequential lentiviral transduction to express CD64/16A and an IL-15/IL-15Rα fusion (IL-15RF) protein to eliminate the need for exogenous cytokine stimulation of the derived iNK cells, as previously described (32, 34). Briefly, the iPSCs were differentiated into iCD34 cells then iNK cells using established methodology (34, 35). Differentiated iNK cells were expanded for 2 weeks using irradiated K562-mbIL21–41BBL feeder cells (34, 35) at a 1:2 NK cell-to-feeder cell ratio in B0 media containing; DMEM and Ham’s F-12 (2:1 ratio) (Corning, NY, USA), 10% heat inactivated human AB serum (Valley Biomedical, Winchester, VA, USA), 1% penicillin-streptomycin (Gibco), 20 μM β-mercaptoethanol (MilliporeSigma, Burlington, MA, USA), 10 μg/ml ascorbic acid (MilliporeSigma), 1.5 ng/ml sodium selenite (MilliporeSigma), 50 μM ethanolamine (MilliporeSigma), and 10 mM HEPES (Gibco, Waltham, Massachusetts, USA). Raji Burkitt lymphoma cells were purchased from American Type Culture Collection (Manassas, Virgina, USA) and were maintained in RPMI- 1640 media (Gibco) supplemented with 10% FBS (Gibco) and 1× pen–strep (Gibco). Raji cells stably expressing firefly luciferase (Raji-Luc), NucLightGreen (NLG), or NucLightRed (NLR) (Sartorius, Gottingen, German) were generated as previous described (36). Cells were routinely tested for Mycoplasma with the MycoAlert Mycoplasma Test Kit (Lonza, Basel, Switzerland).

Peripheral blood-derived NK (PBNK) cells were obtained from whole blood, collected from healthy consenting adults at the University of Minnesota (IRB protocol # 9708M00134) in sodium heparin tubes (BD Bioscience, Franklin Lakes, NJ, USA), and from plateletpheresis products from Innovative Blood Resources (St. Paul, MN). Briefly, peripheral blood mononuclear cells were isolated using Lymphocyte Separation Medium (Corning) per the manufacturer’s instructions. NK cells were enriched using a negative selection human NK Cell Isolation Kit (Miltenyi Biotec, Bergisch, Germany). Isolated NK cells were >95% pure, as determined by CD56^+^ CD3^−^ staining for flow cytometry. PBNK cells were expanded for 2 weeks using irradiated K562-mbIL21–41BBL feeder cells (34, 35) at a 1:2 NK cell-to-feeder cell ratio in B0 media containing; DMEM and Ham’s F-12 (2:1 ratio) (Corning), 10% heat inactivated human AB serum (Valley Biomedical, Winchester), 1% penicillin-streptomycin (Gibco), 20 μM β-mercaptoethanol (MilliporeSigma), 10 μg/ml ascorbic acid (MilliporeSigma), 1.5 ng/ml sodium selenite (MilliporeSigma), 50 μM ethanolamine (MilliporeSigma), and 10 mM HEPES (Gibco). Cryopreservation was performed using CryoStor CS10 Cell Freezing Medium (STEMCELL Technologies, British Columbia, Canada) at 1x10^7^ to 2.5x10^7^ cells per ml in cryogenic vials. Vials were placed in freezer storage containers with isopropyl alcohol for controlled rate freezing at -80°C for 24 hours then moved to liquid nitrogen for storage. Recovery of cryopreserved cells was performed by floating a vial in a 37°C water bath for 1 minute until partially thawed. Cells were centrifuged at 300×g for 5 minutes and resuspended in pre-warmed X-VIVO 15 (Lonza) for experiments unless otherwise noted.

### Flow cytometry

NK cell phenotypic analyses were performed as previously described (32). Briefly, fluorescence minus one (FMO) and appropriate isotype-matched antibodies were used for controls. An FSC-A/SSC-A plot was used to set an electronic gate on leukocyte populations, and an FSC-A/FSC-H plot was used to set an electronic gate on single cells. Antibodies used for the detection of CD64 (clone 10.1), CD3ε (clone UCHT1), CD56 (clone HCD56), CD107a (clone H4A3), and CD16 (clone 3G8) were obtained from BioLegend (San Diego, CA, USA). Therapeutic mAbs used were rituximab (manufactured by Genentech, South San Francisco, CA, USA), obinutuzumab, loncastuximab, and afucosylated rituximab were obtained from InvivoGen (San Diego, California, USA). Appropriate isotype-matched antibodies were used as controls and live/dead discrimination using 7AAD per the manufacturer’s instructions (Biolegend). All immunophenotypic flow cytometry was performed using an FACSCelesta (BD Biosciences), and data was analyzed using FlowJo software (BD Biosciences).

### NK cell antibody arming

iNK-CD64/16A cells and PBNK cells were armed with therapeutic mAbs at a density of 5x10^6^ cells/ml with 5 μg/ml of antibody in serum-free X-VIVO 15 media for 1.5 hours at 37°C and 5% CO2. Cells were then washed extensively with X-VIVO 15 media to remove unbound antibody. For some assays, therapeutic antibodies were biotinylated using the EZ-Link Sulfo-NHS-Biotin Kit (ThermoFisher, Waltham, MA, USA) according to manufacturer’s protocol. iNK cells and PBNK cells were armed with biotinylated antibodies as described above and bound antibody confirmed by staining with fluorophore-conjugated streptavidin and evaluated by flow cytometry, as previously described (32).

### Analysis of NK cell CD107a upregulation in whole blood

CD107a expression during co-culture with Raji cells in whole blood was examined as described by Kim, et al. (37). Briefly, iNK-CD64/16A cells were armed with the indicated antibodies for 1.5 hours and co-cultured with Raji cells in 75% whole blood from a healthy donor at an E:T ratio of 1:1, 3x10^5^ total cells/well, at 37°C and 5% CO_2_. CD107a antibody was added immediately, 2 nM of monensin (BD Biosciences) was added after 1 hour, and cells were incubated an additional 4 hours with gentle shaking at 37°C and 5% CO_2_. Following co-culture, the cells were stained, red blood cell lysis performed with AKC lysing buffer (Quality Biological, Gaithersburg, MD, USA), and analyzed by flow cytometry. Degranulated iNK-CD64/16A cells were identified as CD3^-^, CD56^+^, CD64^+^ and CD107a^+^ after doublet discrimination and live/dead exclusion with 7-AAD.

### Tumor cell cytotoxicity assays

To measure cell cytotoxicity in real-time, NLR or NLG Raji cells were plated at a density of 1x10^4^ cells/well on a 96-well flat bottom tissue culture treated plate. iNK cells or PBNK cells were added at the indicated E:T ratios in X-VIVO 15 media at 37°C and 5% CO_2_. Fluorescent images of live cells were obtained hourly for the duration of the assay using an IncuCyte SX3 live cell imaging and analysis system (Sartorius, Gottingen, Germany), as we have previously described (32). Data are presented as double normalized frequency of target cells remaining. ADCC was calculated by subtracting natural cytotoxicity (no antibody) from antibody added.

### Human IgG xenograft model

NSG mice received i.p. administration of GAMMAGARD (Takeda, Lexington, MA, USA), pooled human IgG, and serum levels of human IgG isotypes were quantified as previously described (32). Briefly, 8–12 week old NOD-*scid* IL2Rgamma^null^ (NSG) mice received i.p. administration of 20mg/mouse of GAMMGARD at the indicated time points. Mice were intravenously (i.v.) injected with 1x10^5^ Raji-luciferase cells. Mice were administered iNK-CD64/16A cells (1x10^7^) i.v. in 1x HBSS. Arming of iNK-CD64/16A cells was done prior to cryopreservation. Bioluminescence imaging (BLI) was performed to quantify tumor burden using an *in vivo* imaging system (IVIS) spectrum (PerkinElmer, Waltham, Massachusetts, USA). Imagines were analyzed using Living Imagine Software (PerkinElmer). Mice were monitored by BLI and survival based on mobility and morbidity behavior.

### Statistical analysis

Data are presented as mean +/- standard deviation. Statistical analysis was conducted using Prism software (GraphPad, La Jolla, CA, USA). The statistical test and *post hoc* test are noted in figure legends and significance is indicated as *p<0.05, **p<0.01, ***p<0.001, ****p<0.0001.

## Results

### Generation and ADCC effector function of iPSC-derived NK cells expressing the FcγR fusion CD64/16A

**Figure 1:**
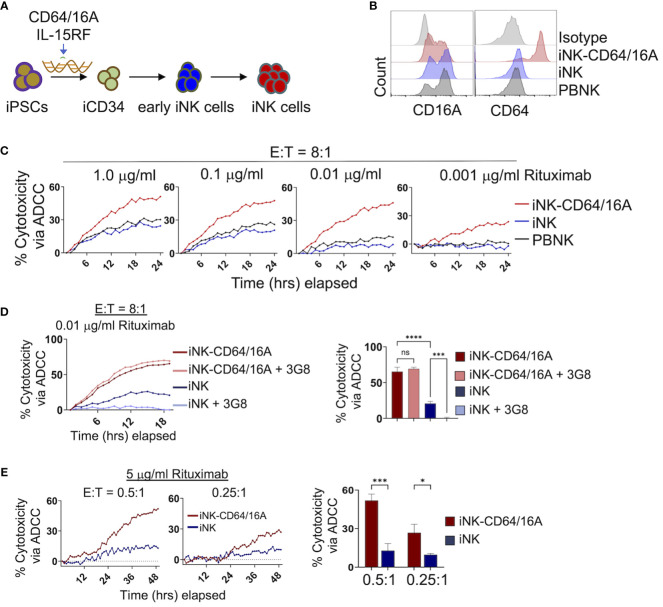
Generation and function of iPSC-derived NK cells expressing CD64/16A. **(A)** Schematic of the genetic engineering of iPSCs and their differentiation to iNK-CD64/16A cells. **(B)** Flow cytometric phenotyping of CD16A and CD64 surface expression on unmodified iNK cells, iNK-CD64/16A cells, and healthly donor PBNK cells following a 2 week expansion with irradiated K562-mbIL21–41BBL feeder cells. Histograms are representative of several independent expansions. **(C)** Unmodified iNK cells, iNK-CD64/16A cells, and PBNK cells co-cultured with Raji cells at an E:T ratio of 8:1 with diluted rituximab, as indicated. **(D)** Unmodified iNK cells or iNK-CD64/16A cells were co-cultured with Raji cells at an E:T ratio of 8:1 in the presence of 0.01 μg/ml of rituximab with or without CD16A blocking mAb 3G8 F(ab′)_2_ (5μg/ml). Percent cytotoxicity via ADCC at 18 hours is graphed (right), n = 3. **(E)** Unmodified iNK cells or iNK-CD64/16A cells co-cultured with Raji cells at the indicated E:T ratios in the presence of 5 μg/ml of rituximab. Percent cytotoxicity via ADCC at 48 hours is graphed (right), n = 3. Statistical significance was determined by ordinary one-way ANOVA with Tukey *post hoc* test. *p<0.05, ***p<0.001, ****p<0.0001, ns = not significant.

iPSCs were genetically modified to express the CD64/16A fusion receptor and membrane-bound IL-15RF, as we have previously described (30, 32, 34). IL-15RF was expressed to support iNK cell activation and expansion without exogenous cytokine stimulation (32, 34). The engineered iPSC cells underwent stepwise differentiation into iCD34 cells, early iNK cells, then final expansion and differentiation by co-culture with K562-mbIL21/41BBL feeder cells (**Figure 1A**). This process generates iNK cells that are phenotypically and transcriptionally similar to PBNK cells (35, 38). CD64/16A expression was assessed by anti-CD64 staining and flow cytometry. iNK-CD64/16A cells stained positively for CD64 whereas unmodified iNK cells did not stain above an isotype control antibodies (**Figure 1B**). PBNK cells from healthy donors were subjected to the same expansion protocol with K562 feeder cells and while CD16A expression was maintained, CD64 was not detected at levels above the isotype control antibody (**Figure 1B**). The ADCC functionality of iNK-CD64/16A cells was evaluated by co-culture with CD20^+^ Burkitt’s lymphoma Raji cells in the presence or absence of the anti-CD20 mAb, rituximab. When serially diluted rituximab was added to the co-culture, rituximab-treated iNK-CD64/16A cells showed markedly higher ADCC activity over a broad range of antibody concentrations (1 – 0.001 μg/ml) compared to PBNK cells and unmodified iNK cells (**Figure 1C**).

We next examined whether enhanced ADCC by iNK-CD64/16A cells was mediated by the fusion FcγR or in combination with endogenous CD16A. To address this, we blocked the function of CD16A with the neutralizing mAb 3G8 (39). A F(ab′)_2_ version of the mAb was used to eliminate confounding effects due to potential Fc binding to high-affinity CD64/16A. Given that PBNK cells and unmodified iNK cells mediated similar levels of ADCC and expressed comparable levels of CD16A, we examined only unmodified iNK cells and iNK-CD64/16A cells under similar co-culture conditions with Raji cells. The greatest difference in ADCC between iNK-CD64/16A and iNK cells occurred at 0.01 μg/ml of rituximab (**Figure 1C**). At this concentration of rituximab, blocking CD16A did not reduce ADCC by iNK-CD64/16A cells, whereas it significantly reduced ADCC by iNK cells (**Figure 1D**), demonstrating that enhanced ADCC by iNK-CD64/16A cells was not due to endogenous CD16A function but rather the expression of the high-affinity fusion CD64/16A. In addition to iNK-CD64/16A cells mediating higher levels of ADCC at lower concentrations of therapeutic mAb, we observed that these cells also mediated significantly higher levels of ADCC at E:T ratios as low as 0.5:1 and 0.25:1 in a 48 hour IncuCyte killing assay (**Figure 1E**). At these ratios, individual NK cells can kill multiple target cells by forming simultaneous lytic synapses or in a sequential/serial manner (40–42). Thus, despite its high-affinity nature, iNK cells expressing CD64/16 may be more efficient at multiple target cell killing by one or both mechanisms than iNK cells expressing endogenous CD16A. Our findings demonstrate that CD64/16A expression by iNK cells markedly enhances their ADCC potency even under more stringent conditions of low E:T and therapeutic mAb concentrations.

### iNK-CD64/16A cells can be armed with mAb to mediate ADCC against lymphoma cells

**Figure 2:**
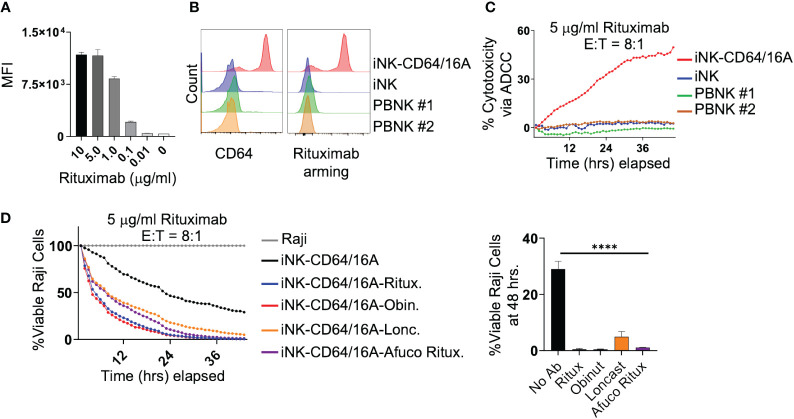
iNK-CD64/16A can be armed with mAbs to mediate potent ADCC. **(A)** iNK-CD64/16A cells were armed with biotinylated rituximab at the indicated concentrations, washed, and stained with a streptavidin fluorophore. Unarmed cells (0 μg/ml) were also stained with streptavidin fluorophore. Cell staining levels were analyzed by flow cytometry. **(B)** iNK-CD64/16A, iNK control cells, or healthy donor PBNK cells were armed with biotinylated rituximab (5 μg/ml), washed, stained with streptavidin fluorophore, and surface stained with CD64 antibody. Cells were gated on CD3- CD56+. Cell staining was analyzed by flow cytometry. **(C)** iNK-CD64/16A, iNK control cells or healthy donor PBNK cells were armed with rituximab (5 μg/ml), washed, and co-cultured with Raji-NLG cells at an E:T ratio of 8:1. Cytotoxicity via ADCC was assessed by live cell imaging. **(D)** iNK-CD64/16A cells were armed with the indicated mAbs, washed, and co-cultured with Raji-NLG cells at an E:T of 8:1. Cytotoxicity was assessed by live cell imaging. The percentage of live target cells remaining are displayed relative to targets alone at time 0. Viable Raji cells at 46 hours is graphed (right), n = 3. Statistical significance was determined by ordinary one-way ANOVA with Tukey *post hoc* test, ****p<0.0001.

An advantage of the CD64 extracellular region of our fusion FcγR is that it binds IgG with approximately 30 to 100-fold higher affinity than CD16A depending on the CD16A allelic variant (28). Our previous work has shown that NK-92 cells expressing CD64/16A can be coupled or ‘armed’ with anti-tumor antigen mAbs (30). We determined that the minimum time for saturation of CD64/16A molecules on iNK cells with human IgG1 antibody was 90 minutes at 37°C (data not shown). Using a similar approach, we determined the minimum concentration of rituximab to saturate CD64/16A on iNK cells at a cell density of 1x10^6^ cells/ml occurred at 5 μg/ml of rituximab (**Figure 2A**). Next, iNK-CD64/16A cells, unmodified iNK cells, and PBNK cells were treated with 5 μg/ml of biotinylated rituximab, extensively washed, and their coupling levels evaluated by flow cytometry. Only iNK-CD64/16A cells demonstrated high levels of antibody arming (**Figure 2B**). All NK cells were then co-cultured with Raji-NLG cells and their lysis was assessed by IncuCyte real-time monitoring. Unmodified iNK and PBNK cells following rituximab coupling did not mediate ADCC, whereas iNK-CD64/16A cells armed with rituximab effectively killed the target cells (**Figure 2C**). Moreover, iNK-CD64/16A cells were significantly more cytotoxic to Raji cells when armed with various anti-CD20 mAb clones including rituximab, afucosylated rituximab, obinutuzumab, as well as the anti-CD19 mAb, loncastuximab (**Figure 2D**). Afucosylated rituximab and obinutuzumab both lack fucose moieties on their Fc region which increases binding affinity to CD16A. These data demonstrate that iNK-CD64/16A cells can be stably armed with assorted therapeutic mAbs irrespective of their fucosylation status to target various antigens, which aligns with previous studies showing that the high-affinity binding of CD64 is not altered by antibody fucosylation status (43).

### Sustained tumor cell killing by antibody-armed iNK-CD64/16A cells

**Figure 3:**
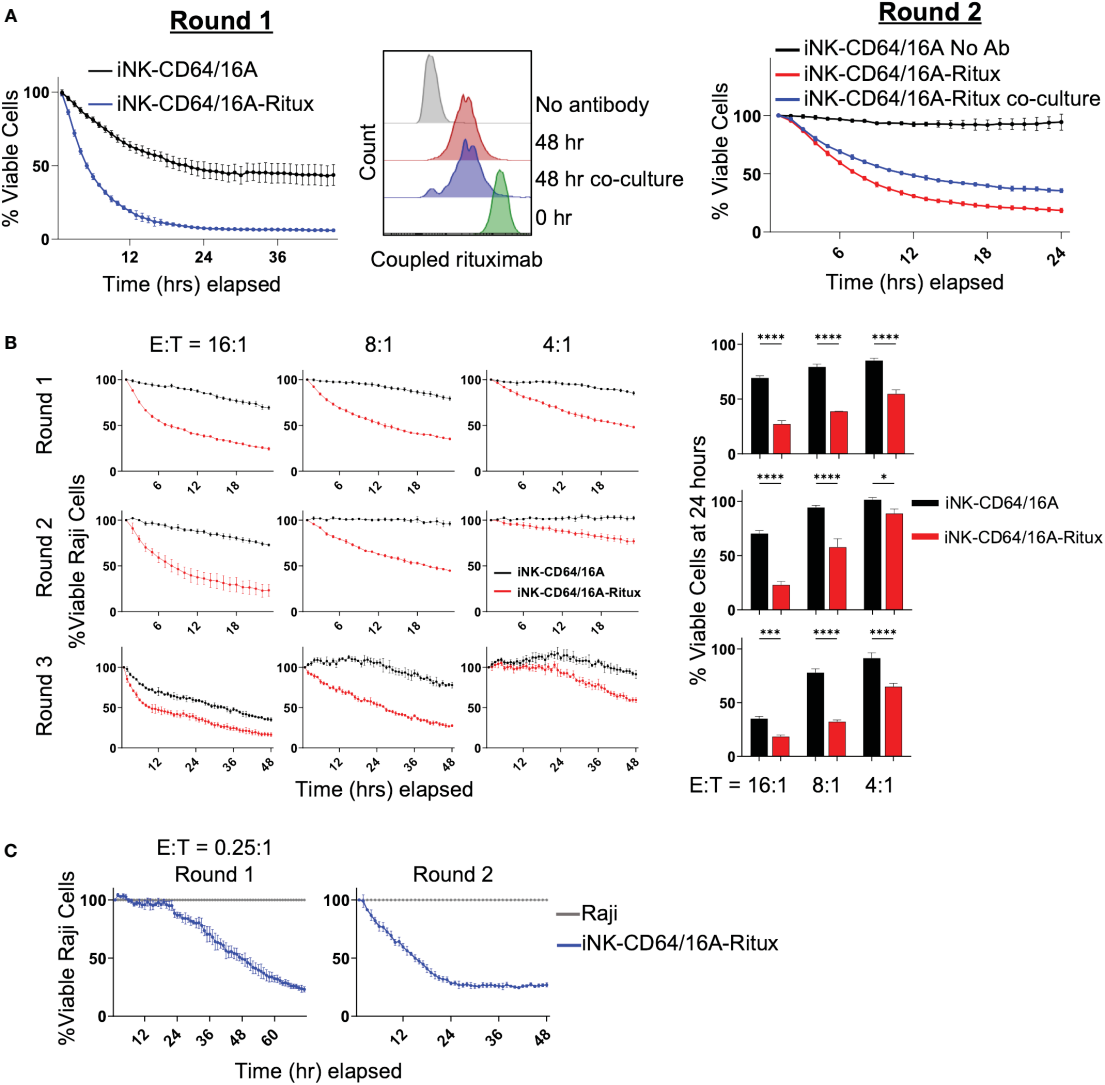
Armed iNK-CD64/16A cells retain tumor-targeting antibody. **(A)** ADCC of Raji cells co-cultured with rituximab-armed (5 μg/ml) or unarmed iNK-CD64/16A at E:T ratio of 10:1 (left, Round 1) or left in the incubator at 37°C for 48 hours, washed, counted, and re-plated for co-culture with Raji cells at E:T ratio of 8:1 for 24 hours (right, Round 2). Staining for rituximab retention was performed following the first 48 hours (middle). **(B)** Rituximab-armed (5 μg/ml) or unarmed iNK cells co-cultured with Raji target cells at the indicated E:T ratios for 24 hours, effector cells were transferred with no adjustment for E:T for an additional two rounds of co-culture. Raji viability at 24 hours for rounds 1 and 2, and 48 hours for round 3 is graphed, n = 3. **(C)** Co-culture of Raji target cells with rituximab-armed (5 μg/ml) iNK-CD64/16A cells at an initial E:T ratio of 0.25:1 over two rounds of killing with no adjustment of E:T for round 2. Statistical significance was determined by paired two-tailed Student’s t-tests. *p<0.05, ***p<0.001, ****p<0.0001.

Next, we sought to determine the persistence of tumor cell killing by antibody-armed iNK-CD64/16A over more than one round of tumor cell co-culture. iNK-CD64/16A cells armed with biotinylated rituximab were co-cultured with or without Raji-NLG cells for 48 hours. Antibody-armed iNK-CD64/16A cells killed Raji cells as expected (**Figure 3A**, left). After 48 hours, biotinylated rituximab-armed NK-CD64/16A cells co-cultured with or without Raji cells were analyzed by flow cytometry to assess the levels of antibody retention (**Figure 3A**, middle). The remaining rituximab-armed iNK-CD64/16A cells from round 1 were washed, counted, and co-cultured with fresh Raji-NLR cells for round 2 (**Figure 3A**, right). Switching from Raji-NLG to Raji-NLR ensured that targets carried over from round 1 were not counted in round 2. ADCC during both rounds was evaluated by IncuCyte monitoring. Armed iNK-CD64/16A cells maintained their ADCC effector function after an initial 48-hour incubation regardless of culture conditions (**Figure 3A**, right). Hence, after round 1, rituximab-armed iNK-CD64/16A cells, whether in the presence or absence of Raji cells, underwent some loss of coupled antibody, but demonstrated sustained ADCC.

To further evaluate the ability of rituximab-armed iNK-CD64/16A cells to undergo sustained ADCC, these cells were co-cultured for 24 hours with Raji cells at E:T ratios ranging from 16:1 to 4:1, and then subsequently transferred, with no readjustment of E:T ratios, to new target cells for a total of 3 rounds of killing. Again, to ensure Raji cells from the previous round were not imaged, Raji-NLG cells were used for rounds 1 and 3, and Raji-NLR cells were used for round 2. At all of the E:T ratios, the rituximab-armed iNK-CD64/16A cells maintained cytotoxicity above unarmed iNK-CD64/16A cells (**Figure 3B**). To better establish whether individual antibody-armed iNK-CD64/16A cells can kill multiple target cells, ADCC assays were performed at the low E:T ratio of 0.25:1 for the first round of killing with no readjustment of the E:T ratio for the second round. Again, the rituximab-armed iNK-CD64/16A cells demonstrated sustained target cell killing (**Figure 3C**). These data indicate that individual iNK-CD64/16A cells can kill multiple tumor targets.

### Antibody-armed iNK-CD64/16A cells can be directed to different tumor antigens to address antigen escape

**Figure 4:**
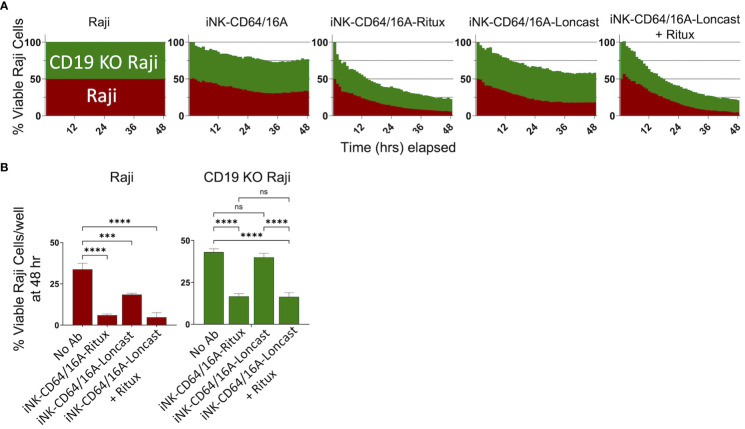
Armed iNK-CD64/16A cells target multiple tumor antigens. **(A)** CD19 and CD20 expressing Raji cells (red) and CD19 knockout (green) Raji cells were mixed in a 1:1 ratio and co-cultured with unarmed or armed iNK-CD64/16A cells for 48 hours at E:T ratio of 8:1. Loncastuximab armed + rituximab had rituximab added to the co-culture at 5 μg/ml. **(B)** Viable Raji cells at 48 hours, n = 3. Statistical significance was determined by ordinary one-way ANOVA with Tukey *post hoc* test. ***p<0.001, ****p<0.0001, ns = not significant.

An advantage to using antibody-armed iNK-CD64/16A cells is the ability to readily switch the antigen targeting element by using different therapeutic mAbs, as shown in **Figure 2D**, unlike the rigid specificity of FDA-approved CAR-mediated antigen targeting. With mAb-armed iNK-CD64/16A cells, multiple antigens can be targeted simultaneously or sequentially (31, 32, 44). We utilized a B cell lymphoma model of antigen escape, as previously described (34). For our assays, we used NLR unmodified Raji cells expressing CD19 and CD20 (Raji) and NLG CD19-deficient Raji cells (CD19 KO Raji) to evaluate the ability of armed iNK-CD64/16A cells to switch tumor antigen targeting. iNK-CD64/16A cells were armed with anti-CD20 rituximab or anti-CD19 loncastuximab and co-cultured with Raji and CD19 KO Raji cells mixed in a 1:1 ratio. Rituximab-armed iNK-CD64/16A cells killed Raji and CD19-deficient Raji cells at levels significantly higher than unarmed iNK-CD64/16A cells (**Figure 4**). Loncastuximab-armed iNK-CD64/16A cells killed unmodified Raji cells, but not CD19 KO Raji cells, at a higher level than unarmed iNK-CD64/16A cells (**Figure 4**). Loncastuximab-armed iNK-CD64/16A cells in the presence of added rituximab, however, were able to kill both Raji and CD19 KO Raji cells similar to rituximab-armed iNK-CD64/16A cells (**Figure 4**). Our data demonstrate the ability of antibody-armed iNK-CD64/16A cells to target distinct tumor antigens, as well as the ability to repurpose these cells by simply administering a new antibody to target an additional antigen.

### iNK-CD64/16A cells mediate ADCC in the presence of serum IgG

**Figure 5:**
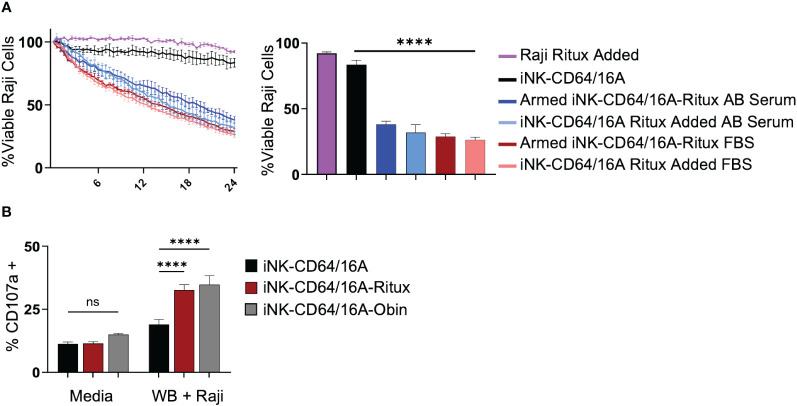
iNK-CD64/16A cells are functional and mediate ADCC in presence of serum IgG. **(A)** iNK-CD64/16A cells were unarmed, armed with 5 μg/ml of rituximab (Armed iNK-CD64/16A-ritux) or had 5 μg/ml or rituximab added to the co-culture in media (iNK-CD64/16A-ritux added) containing 10% FBS or human AB serum against Raji target cells at an E:T ratio of 8:1 for 24 hours. Viable Raji cells at 24 hours are graphed (right), n = 3. **(B)** CD107a expression following co-culture of unarmed, rituximab-armed, or obinutuzumab-armed iNK-CD64/16A cells with Raji targets at an E:T ratio of 1:1 in 75% whole blood for 5 hours. Statistical significance was determined by ordinary one-way ANOVA with Tukey *post hoc* test. n = 3. ****p<0.0001, ns = not significant.

Given the high binding affinity of CD64/16A to IgG, we next sought to determine the competing influence of serum IgG on ADCC *in vitro*. iNK-CD64/16A cells were assessed for their ability to perform ADCC in media containing 10% human AB serum compared to 10% FBS. Data show that there were no significant differences in ADCC by rituximab-armed iNK-CD64/16A cells co-cultured with Raji cells in AB serum or FBS (**Figure 5A**). Moreover, ADCC levels were equivalent for iNK-CD64/16A cells co-cultured with Raji cells in FBS or AB serum when rituximab was added separately (**Figure 5A**), illustrating no significant difference in ADCC between FBS and AB serum-containing media regardless of the method of antibody addition. We next determined the ability of iNK-CD64/16A cells armed with either rituximab or obinutuzumab to mediate ADCC in whole blood. Since the abundance of red blood cells prevented tumor cell imaging in the IncuCyte assay, we evaluated ADCC by assessing CD107a upregulation on iNK-CD64/16A cells during Raji co-culture as a measure of degranulation. CD107a is rapidly transported to the cell surface upon NK cell activation by target cell recognition and a validated surrogate for cytotoxic function (45). When rituximab- or obinutuzumab-armed iNK-CD64/16A cells were co-cultured with Raji targets in “whole blood media” (75% blood plus 25% X-VIVO 15 media), these cells significantly upregulated CD107a when compared to unarmed iNK-CD64/16A cells (**Figure 5B**). Collectively, the above data demonstrate that while CD64/16A binds therapeutic antibodies at high-affinity, high levels of competing IgG did not prevent ADCC by iNK-CD64/16A cells when armed with a therapeutic antibody or directed to an antigen when the antibody was added to the co-culture.

### iNK-CD64/16A armed with rituximab mediate ADCC *in vivo* using a human IgG xenograft mouse model

**Figure 6:**
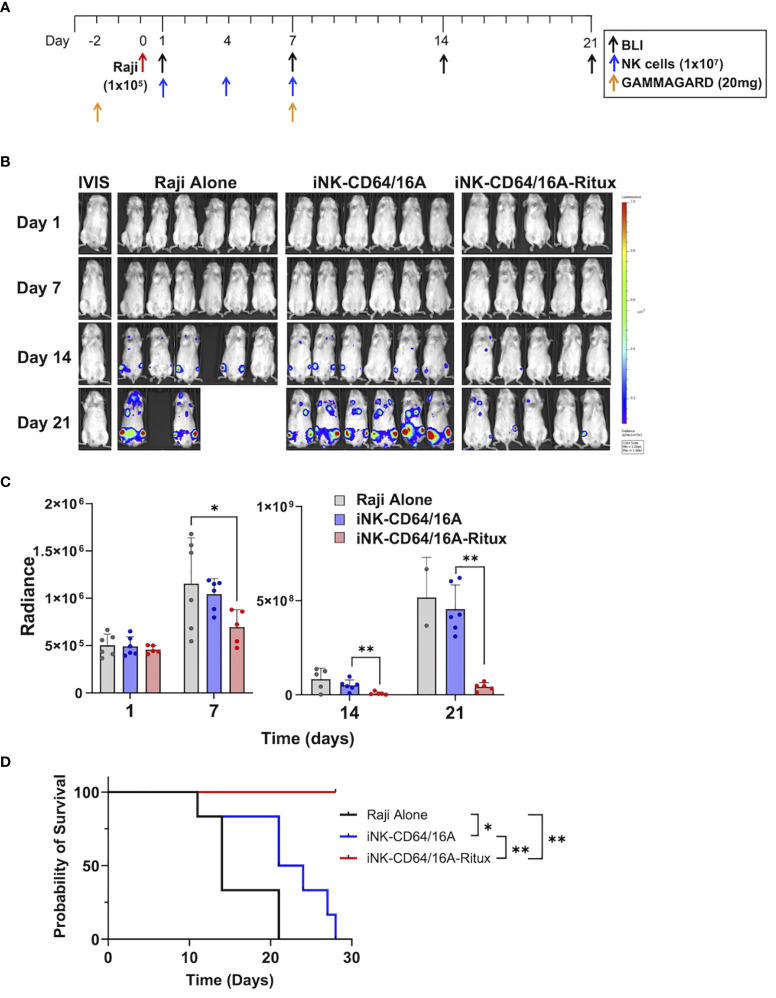
iNK-CD64/16A armed with rituximab mediates ADCC *in vivo* in human IgG xenograft mouse model. **(A)** Treatment schematic. NSG mice were i.v. injected with Raji-Luc cells (1x10^5^); i.p. administered GAMMAGARD (20 mg/mouse); i.v. administered iNK-CD64/16A cells (1x10^7^); and tumor burden was evaluated by BLI, as indicated. **(B)** Bioluminescence images through the first 3 weeks of the experiment. Tumor bearing mice were randomized into three groups: Raji alone (n=6); iNK-CD64/16A (n=6), which received unarmed iNK-CD64/16A cells; and iNK-CD64/16A-rituximab (n=5), which received iNK-CD64/16A cells armed with 5 μg/ml of rituximab. All cells were cryopreserved, thawed, washed, and then administered. GAMMAGARD was administered to all mice. **(C)** Graphical representation of cumulative BLI data. Statistical significance was determined by ordinary one-way ANOVA with Tukey *post hoc* test. **(D)** Kaplan-Meier curves for the *in vivo* Raji experiment. Statistical significance was determined by log-rank (Mantel-Cox) test. *p<0.05, **p<0.01.

To elucidate the capacity for armed iNK-CD64/16A cells to mediate potent ADCC under saturating IgG conditions *in vivo*, we utilized a human IgG xenograft mouse model. Our previous work demonstrated that two doses of 20 mg/mouse of GAMMGARD, a pharmaceutical preparation of human plasma-derived IgG, administered i.p. resulted in near physiological levels of circulating IgG through at least 14 days (32). We used the same IgG dosing, injecting 20mg of GAMMGARD on days -2 and 7 into NSG mice (**Figure 6A**). NSG mice were implanted with 1x10^5^ Raji cells i.v. and then received three subsequent doses of thawed unarmed or rituximab-armed iNK-CD64/16A cells (**Figure 6A**) to test an “off-the-shelf” adoptive cell transfer approach. The Raji and iNK doses were consistent with previous studies (32, 34). The iNK-CD64/16A cells were armed with rituximab, and excess antibody thoroughly washed away, prior to cryopreservation. Tumor burden was determined weekly by BLI. Mice that received rituximab-armed iNK-CD64/16A cells showed a significantly lower tumor burden (**Figures 6B, C**), which was associated with increased survival (**Figure 6D**), compared to mice administered Raji cells and unarmed iNK-CD64/16A cells. These data demonstrate that armed CD64/16A cells can also mediate potent ADCC *in vivo* even in the presence of saturating human IgG.

## Discussion

We evaluated the therapeutic potential of engineered iNK cells expressing CD64/16A as an “off-the-shelf” cellular therapy. Our study demonstrated their flexibility in antibody usage and multi-tumor antigen targeting to treat lymphoma. iNK-CD64/16A cells were more effective at tumor cell ADCC when compared to similarly expanded PBNK cells and unmodified iNK cells, especially at lower E:T ratios and therapeutic antibody concentrations. Moreover, unlike PBNK cells and unmodified iNK cells, iNK-CD64/16A cells could be armed with a therapeutic mAb and mediate potent ADCC, even in the presence of saturating human IgG during *in vitro* and *in vivo* conditions. Further, iNK-CD64/16A cells are potent mediators of ADCC following cryopreservation. The *in vivo* studies utilized iNK-CD64/16A cells which were armed and washed prior to cryopreservation. Our data demonstrates that iNK-CD64/16A can mediate ADCC with soluble or armed mAbs.

Current FDA-approved CAR T cell therapy has shown promising benefits for cancer immunotherapy; however, it is limited in part by donor T cell availability, cell numbers available for infusion, and single antigen targeting. Indeed, patients given anti-CD19 CAR T therapy have a significant CD19-negative relapse rate (12, 15, 46). Thus, better treatments targeting multiple tumor ligands and antigens are needed to address this key issue of antigen escape. As demonstrated here, iNK-CD64/16A cells can be armed with various therapeutic antibodies as well as repurposed with newly added antibodies to the same or additional antigens, offering additional flexibility in antigen targeting, as well as the ability of these cells to be utilized for assorted malignancies. Indeed, therapeutic antibodies offer an ever-expanding arsenal of tumor targeting agents (47).

Our goal is to enhance ADCC by NK cell therapies. This is exclusively mediated by CD16A, a low affinity IgG FcR that undergoes rapid shedding from the cell surface by ADAM17 under various conditions, including cryopreservation, activation, proliferation, and by ADAM17 induction in the tumor microenvironment (20). The polymorphic variant of CD16A with amino acid phenylalanine (F) at position 158 binds to IgG with 2-fold lower affinity than CD16A with a valine (V) in that position (28). Notably, the majority of the population express the CD16A 158F variant (29, 48, 49). Studies have revealed increased efficacy by tumor-targeting antibody therapies in patients expressing the high-affinity polymorphism of CD16A (29, 50, 51), including the anti-CD20 antibody rituximab (29).

With this knowledge, the Fc region of antibody therapies are being modified for CD16A to bind with higher affinity. One modification is afucosylation of the Fc region, which has been shown to increase ADCC and IFNγ generation by NK cells (52–56). However, such antibody modifications then need to be performed on each individual therapeutic mAb, increasing production costs. Moreover, afucosylation and related antibody modifications do not address CD16A shedding upon NK cell activation during ADCC, which greatly decreases antibody binding avidity (24). Interestingly, a study found that afucosylated antibodies induce more CD16A shedding from the cell surface than unmodified antibodies (53). Approximately 6% of endogenous serum IgG is afucosylated (57, 58), and thus these higher affinity IgG molecules compete with unmodified and afucosylated mAb therapies in occupying CD16A. This would not be the case for CD64/16A as afucosylated IgG does not bind to CD64 with higher affinity than unmodified IgG (59). The higher antibody binding affinity by CD64/16A allows for iNK cells to be armed prior to cryopreservation and maintain antibody on the surface to successfully mediate ADCC following thawing and infusion. Since CD64/16A binds IgG with high-affinity, this alleviates the need to modify the antibody, and it is not cleaved by ADAM17 (30), addressing critical hurdles to improving ADCC. Moreover, iNK cells can be clonally expanded for uniformity, and large cell numbers produced for multiple infusions, which is a limitation of current CAR T therapy strategies. In summary, iNK-CD64/16A cells provide a flexible “off-the-shelf” platform for multi-antigen targeting as a therapeutic approach to overcome cancer relapse due to antigen escape.

## Data availability statement

The raw data supporting the conclusions of this article will be made available by the authors, without undue reservation.

## Ethics statement

The studies involving humans were approved by University of Minnesota, Internal Review Board. The studies were conducted in accordance with the local legislation and institutional requirements. The participants provided their written informed consent to participate in this study. The animal study was approved by University of Minnesota, Institutional Animal Care and Use Committee. The study was conducted in accordance with the local legislation and institutional requirements.

## Author contributions

KJD: Investigation, Formal analysis, Methodology, Writing – original draft, Writing – review & editing, Funding acquisition. KMS: Investigation, Funding acquisition, Writing – review & editing. MK: Resources, Writing – review & editing. RH: Formal analysis, Methodology, Writing – review & editing. ZD: Resources, Writing – review & editing. AM: Methodology, Writing – review & editing. SS: Resources, Methodology, Writing – review & editing. BH: Resources, Methodology, Writing – review & editing. RB: Resources, Methodology, Writing – review & editing. MH: Resources, Methodology, Writing – review & editing. JSM: Resources, Methodology, Funding acquisition, Writing – review & editing. BV: Resources, Methodology, Writing – review & editing. JW: Resources, Methodology, Funding acquisition, Writing – review & editing. BW: Project administration, Writing – original draft, Writing – review & editing, Funding acquisition.
